# Risk of placenta previa in second birth after first birth cesarean section: a population-based study and meta-analysis

**DOI:** 10.1186/1471-2393-11-95

**Published:** 2011-11-21

**Authors:** Ipek Gurol-Urganci, David A Cromwell, Leroy C Edozien, Gordon CS Smith, Chidimma Onwere, Tahir A Mahmood, Allan Templeton, Jan H van der Meulen

**Affiliations:** 1 Department of Health Services Research and Policy, London School of Hygiene and Tropical Medicine, London, UK; 2 Office for Research and Clinical Audit, Lindsay Stewart R&D Centre, Royal College of Obstetricians and Gynaecologists, London, UK; 3 Maternal and Fetal Health Research Centre, Manchester Academic Health Sciences Centre, Manchester, UK; 4 Department of Obstetrics and Gynaecology, Cambridge University, Cambridge, UK

## Abstract

**Background:**

Objective: To compare the risk of placenta previa at second birth among women who had a cesarean section (CS) at first birth with women who delivered vaginally.

**Methods:**

Retrospective cohort study of 399,674 women who gave birth to a singleton first and second baby between April 2000 and February 2009 in England. Multiple logistic regression was used to adjust the estimates for maternal age, ethnicity, deprivation, placenta previa at first birth, inter-birth interval and pregnancy complications. In addition, we conducted a meta-analysis of the reported results in peer-reviewed articles since 1980.

**Results:**

The rate of placenta previa at second birth for women with vaginal first births was 4.4 per 1000 births, compared to 8.7 per 1000 births for women with CS at first birth. After adjustment, CS at first birth remained associated with an increased risk of placenta previa (odds ratio = 1.60; 95% CI 1.44 to 1.76). In the meta-analysis of 37 previously published studies from 21 countries, the overall pooled random effects odds ratio was 2.20 (95% CI 1.96-2.46). Our results from the current study is consistent with those of the meta-analysis as the pooled odds ratio for the six population-based cohort studies that analyzed second births only was 1.51 (95% CI 1.39-1.65).

**Conclusions:**

There is an increased risk of placenta previa in the subsequent pregnancy after CS delivery at first birth, but the risk is lower than previously estimated. Given the placenta previa rate in England and the adjusted effect of previous CS, 359 deliveries by CS at first birth would result in one additional case of placenta previa in the next pregnancy.

## Background

Placenta previa can have serious adverse consequences for both mother and baby, including an increased risk of maternal and neonatal mortality[1-3], fetal growth restriction and preterm delivery[4], antenatal and intrapartum hemorrhage[5-7], and women may require a blood transfusion[8] or even an emergency hysterectomy. It is a relatively uncommon condition, with an overall incidence in England of 6.3 per 1000 births[9], but incidence rates are higher among women with advanced maternal age, multiple gestation, high parity, or who smoke or use illegal drugs[10]. The risk of placenta previa is also reported to be higher among women with previous uterine surgery, including cesarean section[11].

In England, cesarean sections constituted 25% of National Health Service (NHS) deliveries during 2010, and the rates have been rising for both primary and emergency CS[9]. The risk of placenta previa in a pregnancy after a CS delivery has been reported to be between 1.5 and 6 times higher than after a vaginal delivery. A meta-analysis of studies published before 2000 of previous CS as a risk factor for placenta previa found an overall odds ratio of 2.7 [10]. However, the overall odds ratio was lower in studies that had better adjustment for confounders[10]. A recent study from the USA that was not included in the meta-analysis, and which used a population-based cohort of 11 million pregnancies, found an adjusted odds ratio of 1.8 for all pregnancies[12] and an adjusted odds ratio of 1.5 for second births only[13].

Evidence about the risk of placenta previa following a previous CS in UK women is limited to results published 25 years ago[14]. We used the Hospital Episode Statistics (HES), an administrative database of all admissions to NHS hospitals in England, to define a population-based cohort and to quantify the association between CS at first birth and the risk of developing placenta previa in the subsequent pregnancy. We also performed a meta-analysis of the reported results in peer-reviewed articles since 1980.

## Methods

### The cohort study

We used the HES dataset for nine financial years from April 2000 to February 2009 for the cohort study. In HES, individual patients are allocated the same identifier for each episode of care[9] and core fields contain patient demographics, clinical information, and hospital administrative data. Diagnostic information is coded using the International Classification of Diseases 10^th ^revision (ICD10) and operative procedures are coded using the UK Office for Population Censuses and Surveys classification, 4^th ^revision (OPCS4). For delivery episodes, the HES dataset also has an additional "maternity tail" which includes parity, birth weight, gestational age, method of delivery, and pregnancy outcome. However, not all records that describe a delivery episode have data entered into this tail.

All women who gave birth to a singleton first and second baby from 1^st ^April 2000 to 28^th ^February 2009 were eligible for inclusion. A delivery was defined as an episode of care that included a relevant OPCS4 code (R17-R25) or ICD10 code (O80-O84) for the mode of delivery, or a maternity tail with a valid date of birth for a baby. Deliveries were coded as a cesarean section by the relevant OPCS4 codes (R17 for primary CS or R18 for emergency CS) or if OPCS4 codes were not available, by the delivery method specified in the maternity tail. If neither of the sets of codes were available (0.5% of delivery episodes), vaginal delivery was assumed.

We confined the analysis to NHS trusts (acute hospital organizations) that had a reasonable level of data completeness on parity, defined as having parity information in the maternity tail for more than 50% of the delivery episodes in at least seven of the nine years covered in the study. NHS trusts were included if the proportion of nulliparous women in that trust was between 25% and 55% (which corresponded to the overall rate in England and Wales ± 15%[15]) to remove hospitals with poor data quality. Women were allocated to the NHS trusts that existed in February 2009 to take account of previous organizational mergers.

We used the HES patient identifier to trace the second births of those women who had been recorded as nulliparous and who had singleton first births in identified NHS trusts. Diagnostic information for the second birth was taken from the core HES diagnosis fields. Cases of placenta previa were identified by the ICD10 code O44. Pregnancy complications were identified using ICD10 codes O10, O11, O16 for pre-existing hypertension; O13 for gestational hypertension; O14 and O15 for pre-eclampsia and eclampsia; O24.4 and O24.9 for gestational diabetes; and O40 for polyhydramnios.

The effect of previous CS delivery on the risk of placenta previa was estimated using unadjusted and adjusted odds ratios. Multiple logistic regression was used to calculate the odds ratios and the risk of placenta previa adjusted for maternal age (<20, 20-29, 30-39, ≥40), maternal ethnicity (White, Asian, Black and Other), deprivation, inter-birth interval (<1, 1-2, 2-3, 3-4, 4-5, ≥5 years), placenta previa at first birth and indicators for pregnancy complications (i.e., pre-existing hypertension, gestational hypertension including pre-eclampsia and eclampsia, gestational diabetes and polyhydramnios). Deprivation was measured using the 2004 Index of Multiple Deprivation (IMD) rank of the English Super Output Areas. The IMD combines a range of economic, social and housing indicators into a single deprivation score for each small area in England [16]. Categories were defined by partitioning the ranks of the 32,480 areas into quintiles (0-20th percentiles, 20-40th percentiles, etc.) and labeled 1 (least deprived) to 5 (most deprived). Women were allocated a category based on their region of residence. We calculated the number needed to harm using the adjusted estimates from the logistic regression[17].

We examined whether the effect of CS on placenta previa rates was related to the level of other risk factors, such as whether the effect of CS on the placenta previa risk differed between younger and older women. The significance of an interaction term between previous CS and other risk factors was assessed with the likelihood ratio test. All analyses were done in Stata/SE 10.0.

The study is exempt from IRB and UK NREC approval as it involved analysis of an existing dataset comprising information on delivery episodes for women who cannot be identified directly or through the HES patient numbers linked to them.

### Review of literature and meta-analysis

We searched Pubmed, Embase, Web of Science, Cinahl and the Cochrane Library for the period January 1980 to January 2011 to identify studies that examined the relationship between previous cesarean section and placenta previa. The keywords of "placenta previa"/"placenta praevia" and "cesarean"/"caesarean" were searched in MESH headings, titles and abstracts to locate relevant articles. We also checked the references of the selected articles and previous reviews. We only included articles written in English and limited the search to peer-reviewed journals.

We selected studies in which placenta previa was diagnosed or recorded at third trimester or during delivery. If two or more relevant articles used the same data source in overlapping years, we selected the study that adjusted the effect size by age or parity, and if there is still replication, the study with the larger sample size.

Estimated log odds ratios and standard errors of log odds ratios were calculated from raw data presented in the included papers. The meta-analysis was performed using a random-effects model, and summarized the degree of consistency across the study results using the I^2 ^measure, the percentage of total variation across studies that is due to heterogeneity rather than chance[18]. Meta-regression was used to assess whether the effect size was associated with date of publication, study design (cohort vs. case-control), source of data (population vs. hospital based), method of diagnosis of placenta previa (confirmed at delivery vs. recorded at hospital or birth registry databases), and whether the results were adjusted for age and parity. We also did a subgroup analysis of population-based cohort studies focusing on the association between first-birth cesarean delivery and second-birth placenta previa and compared the magnitude of this association with the results from our cohort.

## Results

### The cohort study

Between April 2000 and February 2009, there were 4,987,245 singleton delivery episodes in 146 English NHS trusts. It was necessary to exclude 76 NHS trusts due to incomplete parity information. This left 2,484,468 (49.8%) singleton delivery episodes in 70 NHS trusts. Of these, 958,882 (38.6%) women were nulliparous and 399,674 had had a second singleton birth by February 2009. The overall rate of placenta previa for the cohort at first birth was 3.6 and at second birth was 5.3 per 1000 births. The proportion of women who had undergone a CS at first birth was 21.5%. The median birthweight at second birth was 3448 grams (Interquartile range: 3110 -3780 grams).

The rate of placenta previa at second birth was 4.4 per 1000 births for women with vaginal delivery at first birth and 8.7 for women with CS at first birth (unadjusted odds ratio = 1.88). We found that this increased risk of placenta previa persisted after other risk factors were taken into account (adjusted odds ratio = 1.60) (Table 1). The strongest association between a maternal characteristic and the risk of placenta previa was for women who had placenta previa at first birth (adjusted odds ratio = 4.77). Women with advanced maternal age, with polyhydramnios, with very short birth intervals of less than one year, and with birth intervals of more than four years also had higher placenta previa risks. Women with pre-existing hypertension had lower placenta previa risks. Using these estimates, we expect that 359 CS deliveries at first birth would result in one additional case of placenta previa at second birth.

**Table 1:** Maternal characteristics and rate of placenta previa (per 1000 births) in second births

	Number (%) of births	Placenta Previa Rate	Unadjusted OR (95% CI)**	Adjusted OR (95% CI)**
**Previous cesarean delivery**			**p < 0.001**	**p < 0.001**
Yes	86055 (21.5)	8.7	1.88 (1.71-2.07)	1.60 (1.44-1.76)
No	313619 (78.5)	4.4	-	-
**Previous placenta previa**			**p < 0.001**	**p < 0.001**
Yes	1429 (0.4)	43.4	7.74 (5.80-10.34)	4.77 (3.55-6.42)
No	398245 (99.6)	5.2	-	-
Maternal age*			**p < 0.001**	**p < 0.001**
<20	15393 (3.9)	1.9	0.56 (0.38-0.82)	0.58 (0.39-0.86)
20-29	191151 (47.8)	3.6	-	-
30-39	184237 (46.1)	7.1	2.01 (1.82-2.22)	1.83 (1.65-2.05)
≥40	8887 (2.2)	13.2	3.60 (2.90-4.46)	3.01 (2.41-3.75)
Ethnicity*			**p = 0.053**	**p = 0.387**
White	275181 (80.4)	5.6	-	-
Asian	42486 (12.4)	4.8	0.86 (0.74-0.99)	1.03 (0.89-1.21)
Black	12158 (3.6)	5.0	0.91 (0.70-1.17)	0.96 (0.74-1.25)
Other	12448 (3.6)	6.7	1.19 (0.96-1.50)	1.22 (0.97-1.52)
Deprivation*			**p < 0.001**	**p = 0.180**
Q1 Most deprived	122039 (30.6)	4.0	-	-
Q2	90740 (22.8)	5.1	1.30 (1.14-1.49)	1.10 (0.96-1.26)
Q3	85913 (21.6)	6.1	1.52 (1.33-1.73)	1.15 (0.99-1.32)
Q4 Least deprived	99403 (25.0)	6.5	1.63 (1.44-1.85)	1.16 (1.01-1.33)
**Pre-existing hypertension**			**p = 0.080**	**p = 0.026**
Yes	5730 (1.4)	3.5	0.69 (0.44-1.08)	0.62 (0.39-0.98)
No	393944 (98.6)	5.4	-	-
Gestational hypertension***			**p = 0.322**	**p = 0.098**
Yes	8464 (2.1)	5.1	0.85 (0.60-1.19)	0.76 (0.54-1.07)
No	391210 (97.9)	5.3	-	-
**Gestational diabetes**			**p = 0.337**	**p = 0.892**
Yes	5586 (1.4)	5.9	1.19 (0.84-1.69)	0.98 (0.69-1.39)
No	394088 (98.6)	5.3	-	-
**Polyhydramnios**			**p = 0.003**	**p = 0.009**
Yes	1985 (0.5)	11.1	2.11 (1.35-3.28)	1.92 (1.23-3.00)
No	397689 (99.5)	5.3	-	-
**Inter-birth interval**			**p < 0.001**	**p < 0.001**
<1	7511 (1.9)	6.4	1.38 (1.02-1.87)	2.08 (1.53-2.83)
1 to 2	120803 (30.2)	4.8	0.92 (0.81-1.03)	1.01 (0.90-1.14)
2 to 3	133834 (33.5)	5.2	-	-
3 to 4	73992 (18.5)	5.5	1.08 (0.95-1.23)	1.08 (0.94-1.23)
4 to 5	35557 (8.9)	6.1	1.19 (1.01-1.40)	1.19 (1.01-1.40)
≥5	27977 (7.0)	6.6	1.29 (1.09-1.53)	1.32 (1.11-1.57)

* Not including missing values (Maternal age n = 6, 0.0%; Deprivation n = 1549, 0.4%; Ethnicity n = 57401, 14.6% of data missing)

** The model was adjusted for all the variables presented in the table. P-values from the likelihood ratio test

*** Gestational hypertension includes pre-eclampsia and eclampsia

Individual interactions with categories for maternal age, ethnicity, deprivation, maternal risk factors and inter-pregnancy interval did not modify the size of the effect of a previous CS on the risk of placenta previa.

### Review of literature and meta-analysis

**Figure 1:**
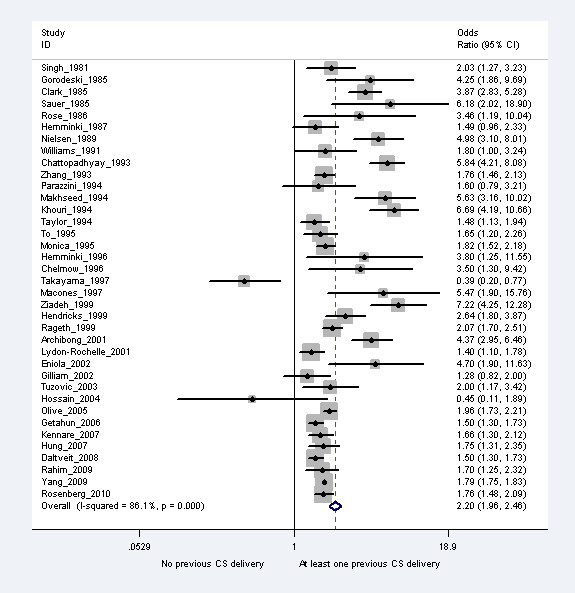
**The relative risk of placenta previa associated with a previous cesarean section**.

We identified 2077 articles of which 74 were of potential relevance to merit a full-text review. 41 articles were selected for inclusion in the review, and an additional eight articles were retrieved from reference lists [8,12,13,19-64]. Among the 49 articles, 12 used overlapping datasets with other studies and were excluded. Study characteristics are presented in Table 2 and the meta-analysis results from the selected 37 studies are given in Figure 1. The overall pooled random effects odds ratio was 2.20 (95% CI 1.96-2.46). The spread of the odds ratios reported in the individual studies were larger than can be expected by chance alone (I^2^= 87.6%).

**Table 2:** Characteristics of the studies included in the meta-analysis

Study ID	Country	Study period	Study design	Population	Size	First two births	Risk adjustment*
Singh_1981	India	1973-1978	Cohort	Hospital	12040	0	-

Gorodeski_1985	Israel	1972-1983	Case-control	Hospital	455	0	-

Sauer_1985	USA	1981-1984	Case-control	Hospital	135	0	-

Clark_1985	USA	1977-1983	Cohort	Hospital	97799	0	-

Rose_1986	England	1978-1982	Case-control	Hospital	160	0	AP;

Hemminki_1987	Sweden		Cohort	Population	7337	1	AP;

Nielsen_1989	Sweden	1978-1987	Cohort	Hospital	24644	0	-

Williams_1991	USA	1977-1980	Case-control	Hospital	12420	0	AP; abortion; alcohol and tobacco use; SE

Chattopadhyay_1993	Saudi Arabia	1988-1992	Cohort	Hospital	41206	0	-

Zhang_1993	USA	1988-1990	Case-control	Population	6478	0	-

Parazzini_1994	Italy	1979-1991	Case-control	Hospital	203	0	-

Makhseed_1994	Kuwait	1992-1992	Cohort	Hospital	8721	0	-

Khouri_1994	Saudi Arabia	1985-1989	Cohort	Hospital	25551	0	-

Taylor_1994	USA	1984-1987	Case-control	Population	1967	0	AP; abortion; tobacco use

To_1995	Hong Kong	1984-1993	Cohort	Hospital	50485	0	-

Monica_1995	Sweden	1983-1990	Case-control	Population	4676	0	AP; previous previa

Hemminki_1996	Finland	1987-1993	Cohort	Population	10889	1	AP; provider

Chelmow_1996	USA	1992-1994	Case-control	Hospital	128	0	-

Takayama_1997	Japan	1974-1994	Case-control	Hospital	264	0	-

Macones_1997	USA	1992-1996	Case-control	Hospital	120	0	age; abortion; drug and tobacco use

Ziadeh_1999	Jordan	1995-1996	Cohort	Hospital	18651	0	-

Hendricks_1999	Singapore	1993-1997	Cohort	Hospital	16169	0	-

Rageth_1999	Switzerland	1983-1996	Cohort	Population	255453	0	-

Archibong_2001	Saudi Arabia	1997-2002	Cohort	Hospital	15191	0	-

Lydon-Rochelle_2001	USA	1987-1996	Cohort	Population	95630	1	AP

Eniola_2002	Nigeria		Case-control	Hospital	272	0	AP; abortion; SE

Gilliam_2002	USA	1986-1989	Case-control	Hospital	1171	1	AP; abortion; tobacco use

Tuzovic_2003	Croatia	1992-2001	Case-control	Hospital	1206	0	-

Hossain_2004	Bangladesh	2000-2002	Cohort	Hospital	2536	0	-

Olive_2005	Australia	1998-2002	Cohort	Hospital	375653	0	-

Getahun_2006	USA	1989-1997	Cohort	Population	156475	1	AP; abortion; alcohol and tobacco use; prenatal care; inter pregnancy interval; SE

Kennare_2007	Australia	1998-2003	Cohort	Population	36038	1	AP; abortion

Hung_2007	Taiwan	1990-2003	Cohort	Hospital	37702	0	-

Daltveit_2008	Norway	1967-2003	Cohort	Population	637497	1	AP; delivery date

Rahim_2009	Pakistan	2001-2005	Cohort	Hospital	20110	0	-

Yang_2009	USA	1995-2000	Cohort	Population	11026768	0	AP; abortion; alcohol, tobacco and drug use; prenatal care; SE

Rosenberg_2010	Israel	1988-2009	Cohort	Hospital	185476	0	AP; abortion; fertility treatment; tobacco use

* AP: age and parity; SE: socioeconomic factors

In bi-variate meta-regressions of various study characteristics on the reported odds ratios (Table 3), significantly smaller effect size of previous cesarean section on placenta previa was associated with recent studies (p = 0.04), population-based studies (p = 0.03), and those that adjusted for age and parity (p = 0.04). The odds ratios reported in the individual studies were not associated with type of study design (cohort vs. case-control) and method of diagnosis of placenta previa (data record in database vs. confirmation at delivery). Heterogeneity across studies remained in subgroup analyses by study design, setting and timing of diagnosis of placenta previa. Results from studies that adjusted for age and parity were less heterogeneous (I^2 ^= 52.3%) and the variation in the results from studies that analyzed first two births were due to chance alone (I^2 ^= 0%).

**Table 3:** Subgroup analysis of the association between previous cesarean section and placenta previa by study characteristics

	Not including current study						Including current study				
	**Number of Studies**	**Random Effects Pooled OR**	**Lower 95% CI**	**Upper 95% CI**	**I^2^**		**Number of Studies**	**Random Effects Pooled OR**	**Lower 95% CI**	**Upper 95% CI**	**I^2^**

**Overall**	**37**	**2.199**	**1.964**	**2.462**	**86.1%**		**38**	**2.155**	**1.941**	**2.395**	**86.0%**
											
**Study design**											
Cohort studies	23	2.345	2.045	2.690	89.7%		**24**	**2.284**	**2.014**	**2.591**	**89.5%**
Case-control studies	14	1.933	1.517	2.464	71.0%		14	1.933	1.517	2.464	71.0%
**Setting**											
Population-based studies	11	1.674	1.549	1.809	86.7%		**12**	**1.664**	**1.553**	**1.785**	**56.0%**
Hospital-based studies	26	2.677	2.146	3.339	86.8%		26	2.677	2.146	3.339	86.8%
**Timing of PP diagnosis**											
Confirmed at CS	13	2.515	1.751	3.613	88.2%		13	2.515	1.751	3.613	88.2%
Other	24	1.982	1.781	2.206	80.9%		**25**	**1.936**	**1.756**	**2.135**	**80.7%**
**Inclusion criteria**											
Studies analyzing 2^nd ^births only	7	1.504	1.383	1.635	0%		**8**	**1.540**	**1.442**	**1.645**	**0%**
Other	30	2.462	2.138	2.835	87.6%		30	2.462	2.138	2.835	87.6%
**Risk-adjustment**											
Adjusted for age and parity	14	1.648	1.515	1.792	52.3%		**15**	**1.642**	**1.524**	**1.769**	**54.1%**
Unadjusted estimates	23	2.712	2.174	3.383	87.7%		23	2.712	2.174	3.383	87.7%

The pooled random effects odds ratio for the six population-based cohort studies analyzing second births only was 1.51 (95% CI 1.39-1.65, I^2 ^= 0%), comparable to our cohort study.

The pooled odds ratio and association of study characteristics on the odds ratio remained similar after inclusion of the current study in the meta-analysis. The subgroup of studies analyzing first two births had significantly smaller effect sizes (p = 0.04).

## Discussion and Conclusions

Among women in England, cesarean section in the first delivery increased the risk of placenta previa in the subsequent delivery by 60%. There was no evidence that the effect of CS on placenta previa rates varied among different groups of women or by the time between two pregnancies. The risks of placenta previa in the second pregnancy also increased by previous placenta previa, advanced maternal age and with birth intervals of less than one year or more than four years. Women with pre-existing hypertension were less likely to have placenta previa. Our results are consistent with recent studies from other countries which typically found odds ratios ranging from 1.4 to 1.7.

### Strengths and limitations of this study

This is the first study of placenta previa risk in England using a large, population-based cohort of nearly 400,000 women with first two births. It included half of all singleton NHS hospital deliveries between April 2000 and February 2009. Due to its comprehensive coverage, the HES database is a valuable resource particularly for studies of relatively uncommon conditions such as placenta previa.

In this study we focused only on the effect of a CS delivery at first birth on a second pregnancy. This has two benefits. First, it corresponds to the information typically required by women and obstetricians. For example, the average achieved family size in the UK is approximately two[65], and so the effect of CS on the second birth is more relevant and accurate than a relative risk based on all pregnancies that may include women with multiple previous CS. Second, estimates based on all pregnancies will be influenced by the proportions of women in 2^nd^, 3^rd^, 4^th^... pregnancies contained in the study sample. Study samples may also differ in the observed sequences of modes of delivery (for example, due to differences in the propensity of local women to opt for trial of labor). Both factors will reduce the comparability of the estimates across studies. We note that, while there is a wide range of reported relative risks, there is consistency in the effect sizes for studies limited to second births.

It is possible that the women with incomplete data in the maternity tail differed from women included in the study in terms of their characteristics and pregnancy risks. However, the distributions of maternal age and mode of delivery were similar in the episodes with good quality data and omitted episodes, which suggests the risk of selection bias is likely to be small.

The coding of the diagnoses and procedures in administrative databases is potentially inaccurate. However, this is unlikely to have had a major effect in our study because previous studies have reported high-levels of agreement in the coding of cesarean section between administrative databases and medical reviews (kappa > 0.98, where stated) [66-68]. Furthermore, cesarean deliveries were defined using the first three characters of the full 4-character OPCS codes, and broader categories rather than specific codes have been shown to be more reliable[69].

Just like other population-based studies, we were not able to confirm the grade and severity of placenta previa [70,71]. We were also not able to control for behavioral risk factors, such as maternal smoking and alcohol and drug use, and previous abortions [40,72-74]. However, the effects of these risk factors as reported in the literature are small compared to the effect of prior CS, maternal age and parity[10].

### Comparison with other studies

In the previous meta-analysis of 21 studies, the pooled odds ratios of previous CS as a risk factor for placenta previa was found to be 2.7 (95% CI: 2.3 to 3.2). The same study emphasized that the odds ratios were highly variable by setting, study design, sample size and quality. For well-designed studies, the pooled odds ratio was 1.9 (95% CI: 1.7 to 2.2)[10].

Since this review, a number of studies have been published. 13 of the 37 studies included in our meta-analysis are post-2000 and cover approximately 12 million women. The pooled odds-ratio of 2.2 (95% CI: 2.0 to 2.5) from this meta-analysis is less than the previous review, and reflects the fact that studies with recent publication dates found smaller effect sizes.

Our results for the English cohort are in agreement with other recent, population-based cohorts analyzing second-births only. In the largest population-based cohort study of over 11 million singleton deliveries between 1995 and 2000 in the USA, the adjusted odds ratio of the effect of previous CS on placenta previa at second-birth was 1.5[75]. Other population-based cohort studies published in the last decade reported adjusted odds ratios ranging from 1.4 to 1.7, using Missouri state birth certificates data[26], Washington state Birth Events data[8], South Australian Perinatal Data[37] and Medical Birth Registry of Norway[49]. A population-based study using data from the Swedish Birth Registry found a higher adjusted odds ratio of 1.8, but this study did not adequately control for risk factors[76].

A few studies have investigated whether the effect of a previous CS on the risk of placenta previa was modified by other risk factors. The Missouri cohort study found that the effect of CS was 70% higher for women with a second pregnancy within a year after the first delivery[26]. We did not find evidence to support the hypothesis that inter-pregnancy interval influences the size of effect of first birth CS. Our results are consistent with the Washington state cohort study that found the size of effect of CS was not influenced by maternal age at second birth [8].

### Implications for policy and practice

Cesarean section rates are rising worldwide, and an increase in the longer term complications of CS should be anticipated. The presumed short and long term safety of CS is probably one of the factors underlying both the growing rate of CS and the wide variation in CS rates not accounted for by case mix. There is a need for better understanding of the relative risks associated with vaginal and CS births to support decision-making by mothers and clinicians[77].

The Confidential Enquiry into Maternal and Child Health recommends that women with a prior CS should have placental localization in current pregnancy to exclude placenta previa[78]. If placenta previa is diagnosed, there must be further investigation to exclude placenta accreta, a potentially life-threatening condition. In high-income countries, advanced radiological facilities can help to diagnose this serious condition in antenatal care and to plan delivery in a tertiary care unit. However, in low or middle-income countries, placental conditions may be encountered first time at the CS with its serious consequences and it is imperative that senior and experienced doctors are involved in the care of women with placenta previa from the outset.

Our study has demonstrated that, in addition to women with previous CS, women with advanced maternal age, women with birth intervals of less than one year and women who had a previous placenta previa are at a higher risk of developing placenta previa. Nonetheless, our study suggests that the absolute risk remains small. Women who had placenta previa in their previous pregnancy were at the greatest risk of placenta previa in a current pregnancy but less than 5 in 100 of women with a previous placenta previa would be expected to have this complication again. Clinicians should consider and communicate these factors when counseling their patients using appropriate and simplified risk statistics[79]. Our study quantifies this risk and provides data that can be used to reassure women, attending for pre-conception counseling clinics or antenatal clinics as well as in pre-operative consent discussions for women undergoing CS.

## Competing interests

The authors declare that they have no competing interests.

## Authors' contributions

All authors have fulfilled all conditions required for authorship. IGU, GCS, LCE, DC, JvdM and TAM designed the study. IGU analyzed the data supported by DC and JvdM. IGU wrote the first draft. All authors contributed to the interpretation of the results, revised further drafts, and approved the final manuscript.

## Pre-publication history

The pre-publication history for this paper can be accessed here:

http://www.biomedcentral.com/1471-2393/11/95/prepub
